# Raw dataset of compression tests on a vegetable oil-based polyurethane foam exposed to different ageing conditions

**DOI:** 10.1016/j.dib.2024.110199

**Published:** 2024-02-15

**Authors:** Enio H. P. Da Silva, Silvio De Barros, Pascal Casari, Marcelo L. Ribeiro

**Affiliations:** a Aeronautical Engineering Department, São Carlos School of Engineering, University of São Paulo, São Carlos, SP 13563-120, Brazil; b CESI LINEACT, Saint-Nazaire 44600, France; c Nantes Université, Ecole Centrale Nantes, CNRS, GeM, UMR 6183, Saint-Nazaire 44600, France

**Keywords:** Compression test, Ageing, Polyurethane, Cellular material, Digital image correlation

## Abstract

The current dataset brings raw compression test information of a vegetable-based polyurethane foam (PUF) exposed to different temperatures over different periods of time. Such experimental dataset can provide researchers with important information in the application of numerical and data-driven simulations. Also, it saves money and time once the experimental part is already available. At total, 90 compression tests were done following the ASTM D1621-16 standard with pictures for digital image correlation (DIC) being simultaneously acquired. The 90 specimens were divided in nine different ageing conditions. The foam was considered transversely isotropic, thus, 10 specimens for each condition were divided in two groups, five specimens for direction 1 and five for direction 3, where direction 3 is the foam expansion direction. The 3D DIC results show longitudinal and transverse strains from virtual extensometers. The results are available in *.TRA* and .*csv* files for the tests and DIC outputs, respectively. Also, the dataset brings the pictures used for DIC in .TIF format. It also brings the dimensions of each specimen prior to the test in *.txt* format. These results provide information for the calculation of major mechanical properties that can be freely used in finite element models for different and creative ways to simulate the ageing process of a vegetable-based PUF.

Specifications Table

**Table:** 

Subject	Material Characterization
Specific subject area	Compression test, accelerated ageing, polymer degradation, polyurethane, digital image correlation
Data format	Raw
Type of data	Table, Graph, Images
Data collection	Compression tests were carried out in a Zwick/Roell Z050 testing machine. The images used in the digital image correlation were taken with two standard cameras from the VIC 3D setup.
Data source location	Institution: GeM - Research Institute in Civil and Mechanical Engineering City: Saint-Nazaire Country: France Latitude and longitude: 47.28, -2.209
Data accessibility	Repository name: Mendeley Data Data identification number: 10.17632/2sp8fyvhfm.2 Direct URL to data: https://data.mendeley.com/datasets/2sp8fyvhfm/3[1]

## 1 Value of The Data

• Researchers in material science, engineering, chemistry, computer science, among others can benefit from mechanical test raw dataset, such as compression tests, either in numerical simulation or machine learning algorithms. Furthermore, the experimental ageing process requires specific storage conditions for long periods of time. Thus, the availability of such data makes the scientific process towards theoretical and computational sciences much faster, which would finally ending up as economical gain.
• The dataset contains raw machine data from compression tests carried out in a bio-based PUF exposed to an accelerated aging process under different temperatures. Thus, in a world that is each day more concerned about lifespan, discard process and carbon footprint of materials, such data can make a bio-based material more interesting to many kinds of applications.
• The foam used to create this dataset was synthesized in a controlled environment in laboratory conditions. Thus, the standardization of the process is assured.
• Therefore, by working on the data, scientist of different fields can better understand how a vegetable-based foam behaves mechanically under different environmental conditions and then, by working on the data, scientist of different fields can better understand how a vegetable-based foam behaves mechanically under different environmental conditions and the accordance of the data with current available models, which can further collaborate for important sustainability progresses.

## 2 Background

It is possible to estimate the ageing process over the mechanical properties of materials by performing mechanical tests in accelerated aged specimens. The most common ageing behaviour is described by Arrhenius equation:

$$K=A\;\text{exp}\left(-\frac{E_{a}}{R\;T}\right)$$

**Fig. 1:**
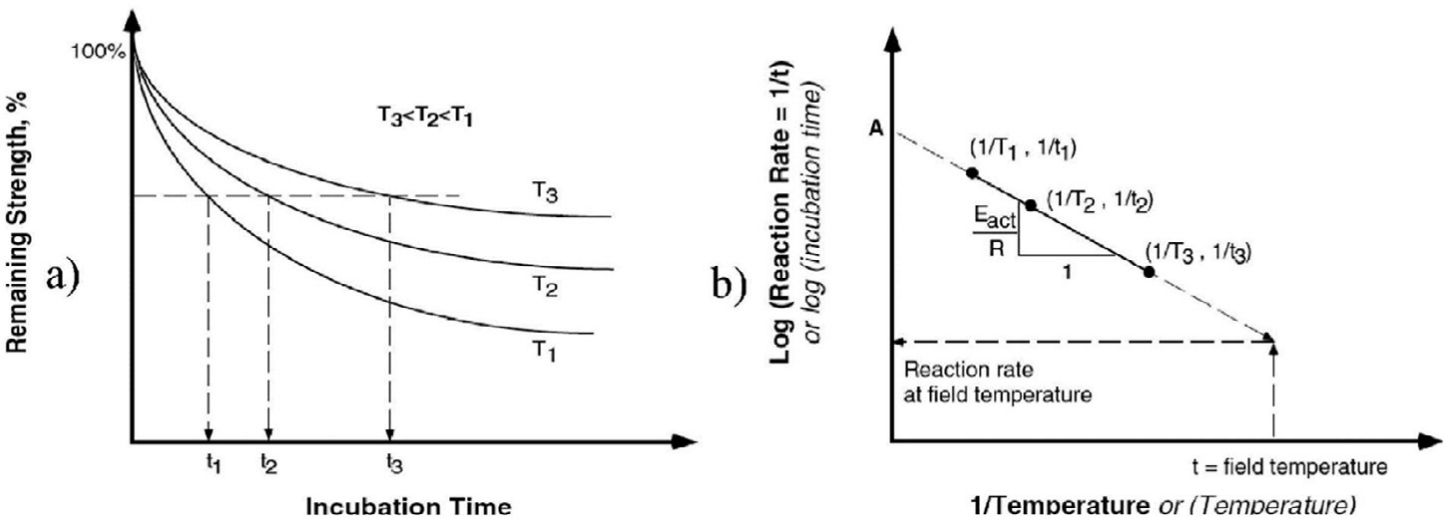
Arrhenius plot [2].

This equation requires specimens aged at three different temperatures in order to normalize their degradation curve and then calculate the activation energy (E_a_), which is represented by the slope of the normalized Arrhenius plot shown in Fig. 1 [2].

In this way, authors have predicted the mechanical behaviour of materials over decades, with short incubation periods, which ends up saving money and time. As the concern for green materials replacing oil-based ones grows, so grows the interest regarding the stability of such materials. This is where this dataset comes in hand, where it will allow researchers to predict the properties of a vegetable-based foam depending on their operation temperature and humidity.

## 3 Data Description

**Fig. 2:**
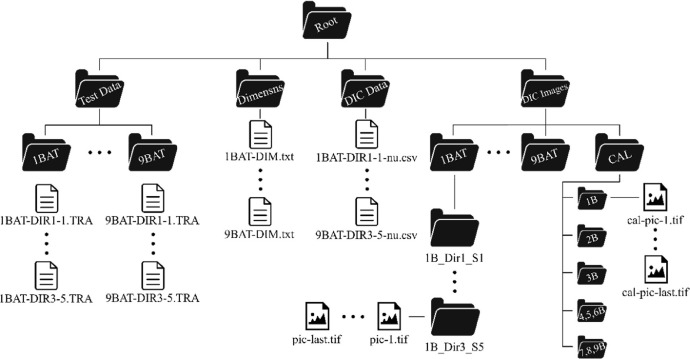
Folder structure.

The root directory contains three folders as shown in Fig. 2. The first one named “Test Data” is divided in 9 subfolders where each one brings 10 raw compression test results in *.TRA* format, these 10 data are divided in two categories, 5 data tested in direction 1 and 5 data for direction 3. Direction 3 represents the direction of expansion in the foaming process and direction 1 represents the transverse direction. The files have three columns, each one for a respective parameter as follows: column 1 is load in *N*, column 2 is displacement in *mm*, and column 3 is time in *s*, the columns are separated by semicolons.

The “Dimensns” folder possesses the information of the dimensions of each specimen for each batch. The files are in *.txt* format and each file contains 3 columns divided by tabulation and 10 rows. The first, second and third columns represent the dimensions in *mm* for the directions 1,2, and 3, respectively. The 10 rows are divided in the following way: rows 1-5 are the five specimens tested in direction 1 and rows 6-10 are the samples tested in direction 3.

The “DIC Data” folder brings the DIC results for most of the tested samples and the results are divided in three columns. The first one represents the index of the data. The second and third columns represent the longitudinal and transverse strains, respectively. The files are in *.csv* format. The analysis of some of the specimens was impossible due to problems with the speckle pattern recognition. Furthermore, the data fully contains strain values in the elastic regime and a significant number in the plastic one, which allows the calculation of the Poisson's ratio in both zones.

The “DIC Images” folder has 10 folders inside, where 9 of them separate the batches and the last one (named “CAL”) has the calibration images. Each batch folder has 10 other folders inside, one for each specimen. The specimens’ folders are named after their batch and direction. As an example, we can use the first specimen tested in direction 1 for the first batch, the name of the folder is going to be 1B_Dir1_S1 (1B = 1^st^ batch, Dir1 = direction 1, and S1 = specimen 1). The images for each test are located inside their respective specimen folder. The name of each picture was given by the VIC-Snap software in .TIF format. The calibration images for the first 3 batches are separated in one folder each. However, since the tests of the batches 4, 5, and 6 were carried out in the same day with no interruption, they all possesses one single set of calibration images that are located in the folder named “4,5,6B”. The same was done with the calibration images of batches 7,8, and 9.

## 4 Experimental Design, Materials and Methods

### 4.1 PUF Synthesis

The material represented in the dataset is a vegetable-based PUF derived from castor oil. This foam is a bi-component polymer and was acquired from Kehl Company. The names of the components appointed by the manufacturer are IC200 and KT1106-R for the isocyanate and polyol, respectively. Furthermore, the manufacturer provides the following pieces of information about the components:

• The isocyanate is a Methylene Diphenyl Diisocyanate (MDI) with a density of 1.22 g/cm3 and viscosity between 170 and 250 mPa·s at room temperature.
• The polyol is a bio-based material from a blend of vegetable oils (mainly castor oil). Its density is 1.0 g/cm3 and its viscosity is around 3000 mPa·s.

**Fig. 3:**
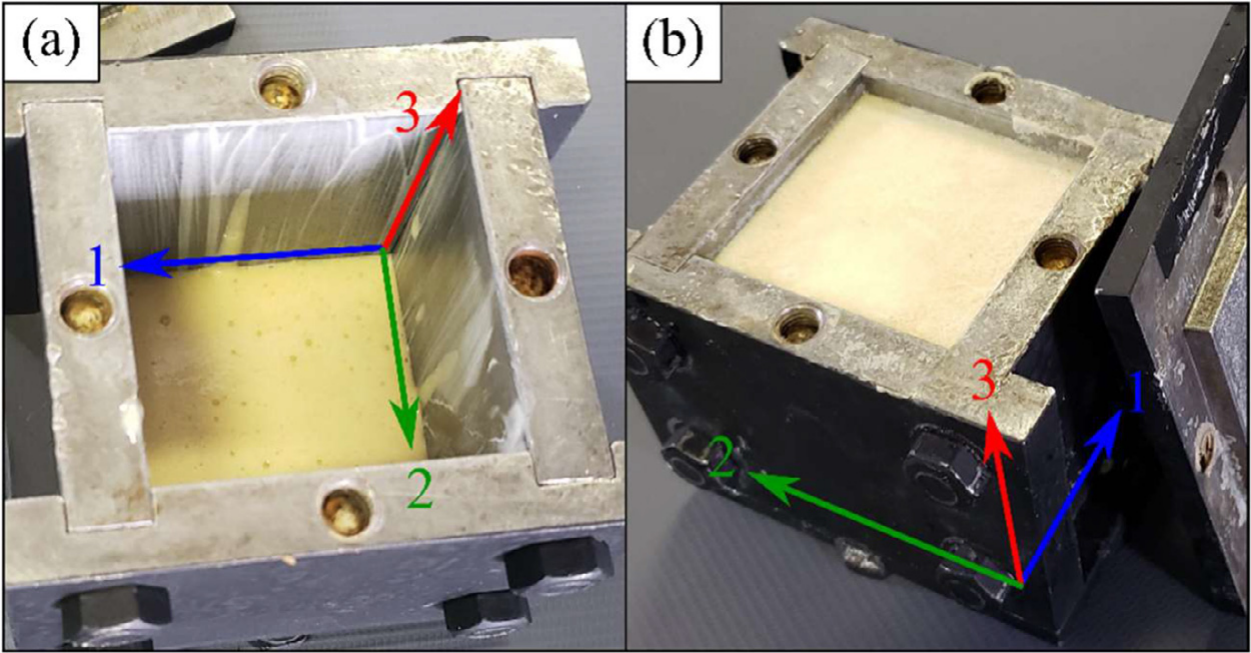
Difference between the directions.

The synthesis was carried out in a fume hood in the GeM laboratory of the IUT of Saint-Nazaire. The components were heated up to 80 °C before homogenization and the components mass ratio in the mixture was 1:1. 9 g of each component (weighted in a precision scale by Kern) was poured in a plastic container followed by a hand-homogenization process. After homogenization, 12±0.5 g of the mixture was poured inside the mold, this number was chosen because some material would remain in the plastic container and in the mixing device. The mold has internal dimension of 50.8×50.8×50.8 mm³. A curing time of 30 minutes at 80 °C was respected. A leakage of around 0.5 g was noted in almost every manufacture process. After the curing time, the foaming expansion direction was marked following the standard shown in Fig. 3, where Fig. 3 (a) shows the mixture in the mold before expansion and (b) after the curing time.

### 4.2 Ageing Process

**Fig. 4:**
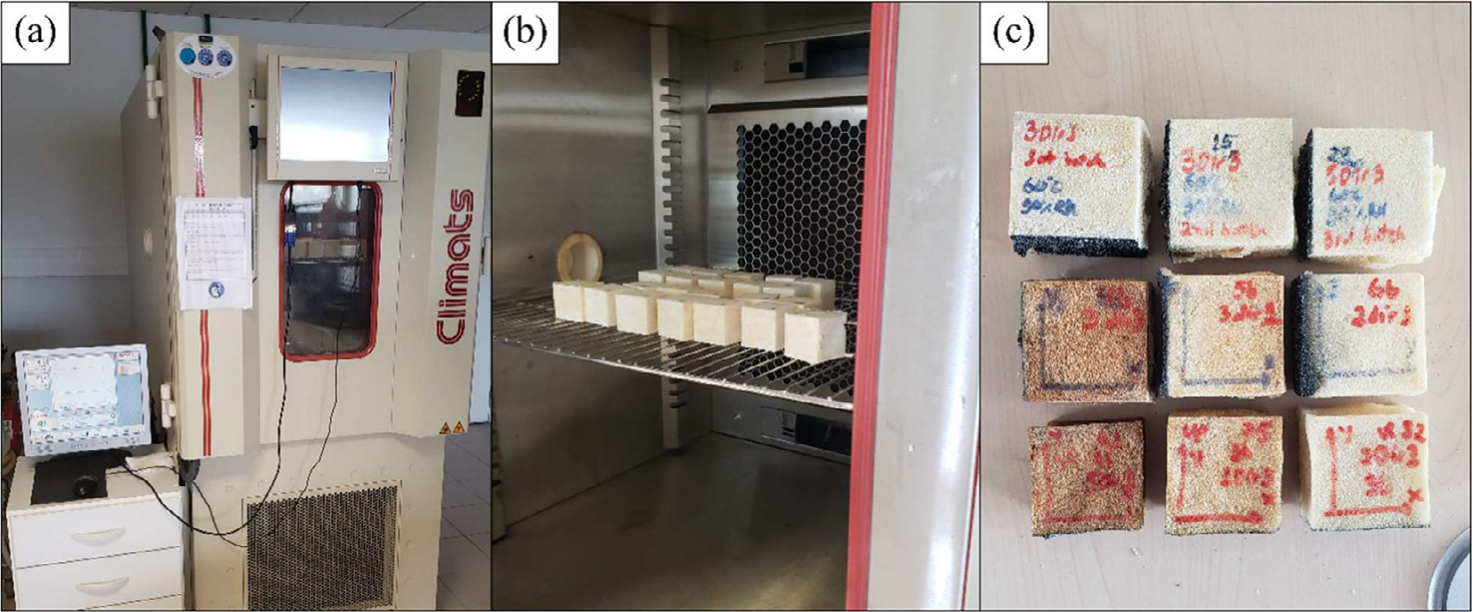
(a) Climate chamber, (b) specimens during the ageing process, (c) specimens after ageing.

After manufacture, the specimens were sanded, weighted, measured and cataloged in the respective order. Then, the accelerated ageing process was carried out in a computer-controlled climate chamber by Climats. The specimens and chamber are shown in Fig. 4.

The chosen temperature range was based on previous studies with polyurethanes from different authors [3], [4], [5] and is shown in Table 1 along with the time the samples were left in chamber. Furthermore, the data shows the ageing in those temperatures in the presence of a relative humidity of 90%, that besides the increased amount of oxygen causing polymer oxidation, also damages the mechanical properties by water diffusion and erosion [6,7].

**Table 1:** Ageing conditions for each batch os samples.

Batch number	Temperature (°C)	Relative humidity (%)	Time (days)
1	60	90	25
2	60	90	35
3	60	90	60
4	75	90	10
5	75	90	30
6	75	90	55
7	90	90	5
8	90	90	10
9	90	90	15

### 4.3 Compression Test

**Fig. 5:**
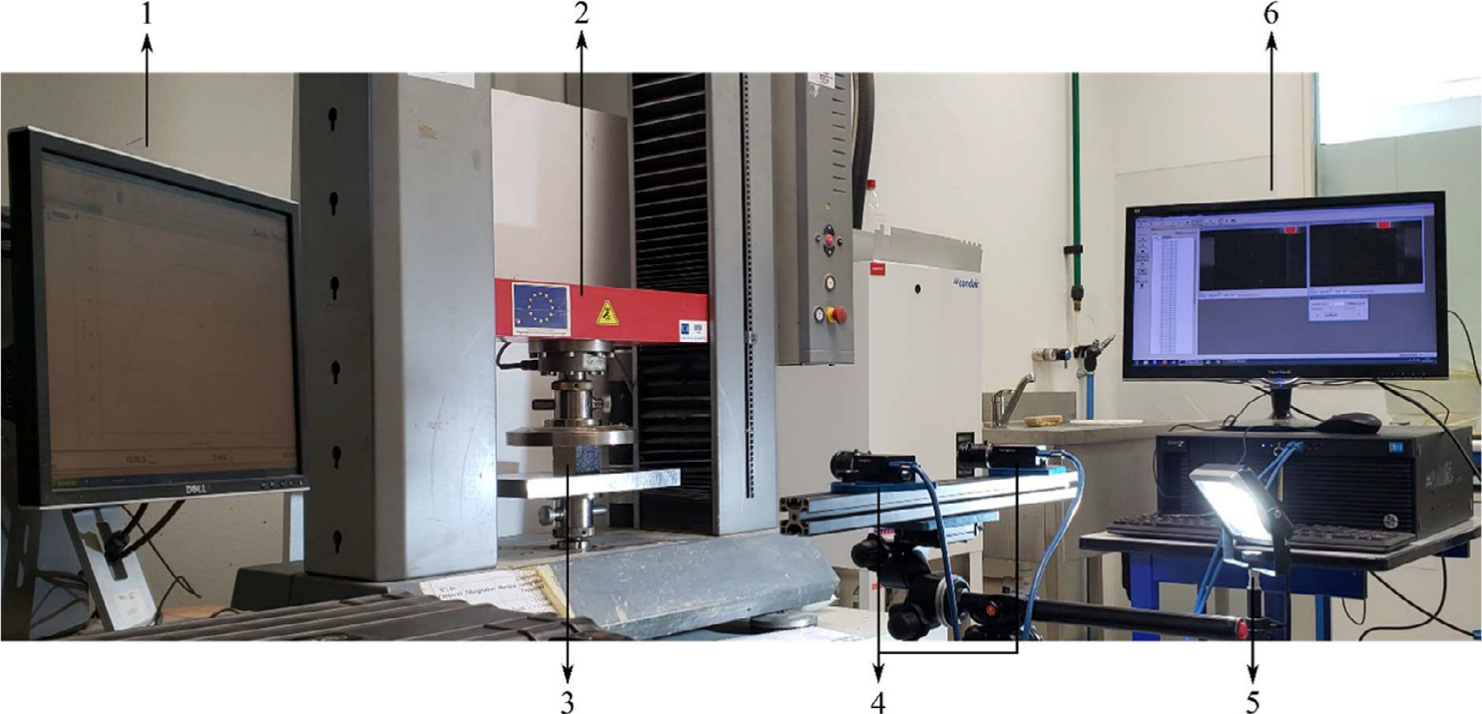
Compression test scheme where item 1 is the computer controlling the UTM, 2 is the crosshead of the UTM, 3 is the test specimen, 4 is the cameras for the 3D DIC, 5 is the lightning, and 6 is the VIC-Snap software taking pictures for the DIC.

The compression tests were carried out in a Zwick/Roell Z050 universal testing machine (UTM). The data was directly obtained from its software *TestXpert II* every 0.1 s. The test followed the standard ASTM D1621-16 [8]. This standard has been widely used in numerous scientific works in the last years [4,[9], [10], [11]]. The test speed was set to 5 mm/min. Also, according to the standard, the specimen dimensions were prepared as 50×50×50 mm³ before the ageing process. However, after ageing, there was a significant dimensional changing and the correct values of the dimensions of the specimens are in folder *DIMENSNS*. Fig. 5 shows the test scheme used in the data acquisition during the compression tests.

### 4.4 DIC Analysis

The speckle pattern applied was an extrinsic one, created by painting a matte black background in all specimens followed by a sprayed white paint that creates white spots over the black background. The white paint speckles had different sizes, ranging approximately from 0.05 mm to 2 mm. Since the painting process was carried out by hand spraying, there was no refined control of the distances between the spots.

**Fig. 6:**
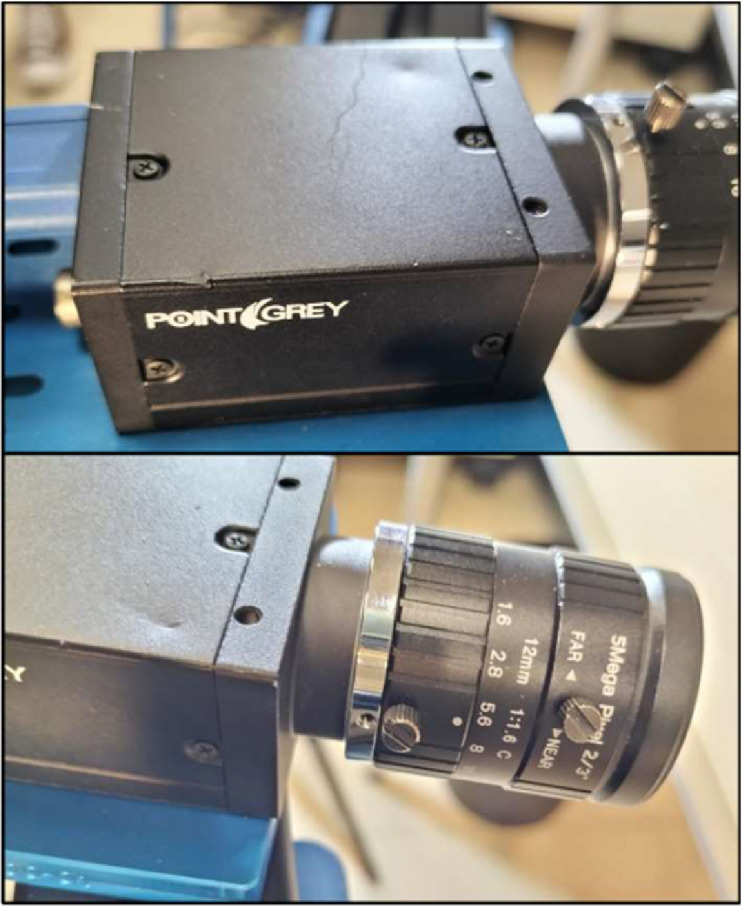
Cameras used to capture the DIC images.

The DIC analysis was carried out in the VIC-3D software, with 2 cameras of 5 Megapixels by PointGrey Inc. with 2/3” optical format and 12 mm focal length, the cameras are shown in Fig. 6. The resolution of the pictures was 1920×1200 pixels, and the field of view (FOV) in the depth of the specimen was around 225 mm.

The system was calibrated with 8 pairs of calibration images that make the software aware of the distances between cameras and specimen. The calibration images were made for different configurations, such as translated and rotated images to fully cover the working volume of the optical system. Vic-3D has a calibration evaluation tool that informs the quality and the proper utilization of the calibration images by providing them a score. The score of the images in these tests were no higher than 0.05, which is acceptable since scores below 0.1 are acceptable for DIC.

**Fig. 7:**
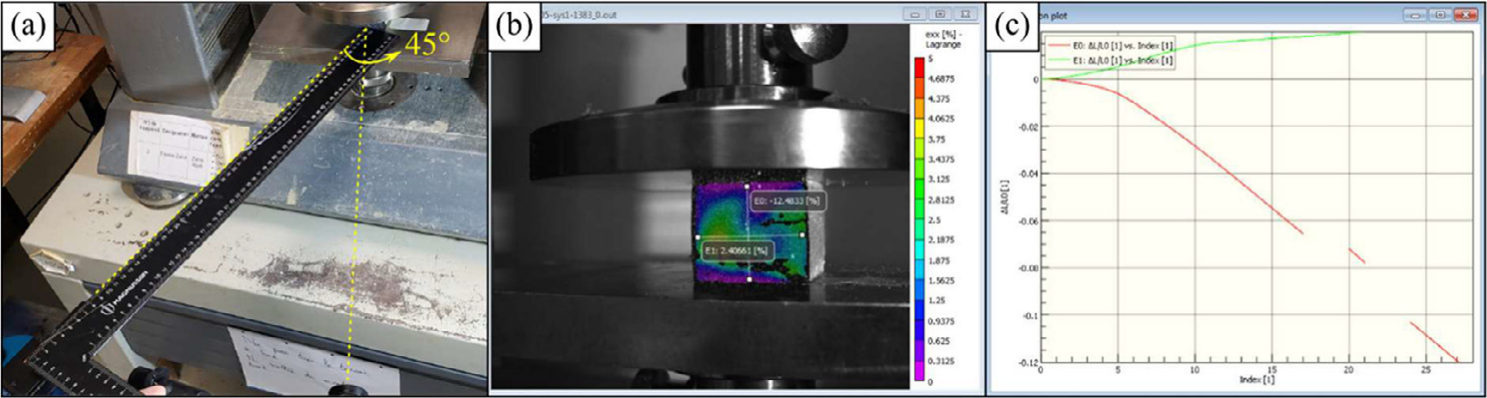
(a) Positioning of cameras, (b) longitudinal and transverse extensometers, (c) strain curves of the extensometers.

The cameras were positioned as shown in Fig. 5 with a distance between lenses of 40 cm and between lens and specimen of 53 cm in order to create a 45° angle between the specimen and the cameras as shown in Fig. 7(a). In the analysis, one virtual extensometer was created for the longitudinal strain and another one for the transverse strain, they were created with facet size of 15 pixels and distance between facets of 10 pixels. The extensometers are shown in Fig. 7(b). The coherence of the data was checked in the strain-image curve shown in Fig. 7(c). The length of the extensometers was different in horizontal and transverse directions, since the top and bottom regions of the specimens are usually affected by shadows and reflection. Therefore, 45 mm and 40 mm were the length for the extensometers in horizontal and transverse directions, respectively, for the first 6 batches. 35 mm and 30 mm were the length for the extensometers in horizontal and transverse directions, respectively, for batches 7, 8, and 9 because the accelerated ageing conditions they were exposed shrunk them. The data was acquired every 3 s after the beginning of the test for the first 3 batches. After realizing that too many pictures were being taken and the same success could be achieved with a larger gap of time between them, an interval of 5 s was adopted for the rest of the batches.

## Limitations

The 3D DIC application requires a precise and refined calibration of the system and even though most of the samples could provide strain data, some of them were not able to provide reliable strain information due to poor calibration prior the test and the DIC data of those specimens were not considered in this dataset.

The DIC technique requires a speckle pattern over the specimen surface. However, when high deformations are applied, such as the ones suffered by the samples in this work, the DIC is not able anymore to recognize the speckle pattern. Therefore, in this work, the strain values were limited to the last strain the VIC-3D software was able to detect.

As shown in Fig. 7(a), the cameras are already pretty close to the base of the testing machine, and as seen in Fig. 7(b), the region of interest does not cover the majority of the FOV. This happened because we have used the lens available in our laboratory to carry the experiments on.

## Ethics Statement

The authors have read and follow the ethical requirements for publication in Data in Brief and confirmed that the current work does not involve human subjects, animal experiments, or any data collected from social media platforms.

## CRediT authorship contribution statement

**Enio  H.  P. Da Silva:** Conceptualization, Methodology, Data curation, Writing – original draft. **Silvio De Barros:** Conceptualization, Supervision, Writing – review & editing. **Pascal Casari:** Resources, Methodology, Supervision, Writing – review & editing. **Marcelo L. Ribeiro:** Project administration, Conceptualization, Supervision, Writing – review & editing.

## Data Availability

Aged PUF compression tests (Original data) (Mendeley Data).
